# Long-term comprehensive cardiopulmonary phenotyping of COVID-19

**DOI:** 10.1186/s12931-022-02173-9

**Published:** 2022-09-21

**Authors:** Lucas M. Kimmig, Zvonimir A. Rako, Stefanie Ziegler, Manuel J. Richter, Ashkan Tolou G.S., Fritz Roller, Friedrich Grimminger, István Vadász, Werner Seeger, Susanne Herold, Khodr Tello, Ulrich Matt

**Affiliations:** 1 Department of Internal Medicine II, Universities of Giessen and Marburg Lung Center, Justus Liebig University, Member of the German Center for Lung Research (DZL), Giessen, Germany; 2 Section of Pulmonary and Critical Care, Department of Medicine, The University of Chicago, Chicago, IL USA; 3 Department of Radiology, University Hospital Giessen, Giessen, Germany; 4 Institute for Lung Health (ILH), Justus Liebig University Giessen, Giessen, Germany; 5 Excellence Cluster Cardio-Pulmonary Institute (CPI), Giessen, Germany; 6 Max Planck Institute for Heart and Lung Research, Bad Nauheim, Germany

**Keywords:** COVID-19, Cardiopulmonary exercise testing, Post-COVID syndrome

## Abstract

**Background:**

Persistent symptoms after initial COVID-19 infection are common and are frequently referred to by the umbrella terms “post-COVID syndrome” and “long COVID”. The sheer number of affected patients pose an increasing challenge to healthcare systems worldwide. To date, our understanding of the pathophysiology of the post-COVID syndrome remains poor and the extent to which persistent cardiopulmonary abnormalities contribute to the symptom complex is unclear. We sought to determine the presence and impact of cardiopulmonary sequelae after COVID-19 in longitudinal assessment.

**Methods:**

We report on 71 patients who underwent comprehensive, longitudinal testing in regular intervals for up to 12 months after their initial COVID-19 diagnosis. Testing included pulmonary function testing, cardiopulmonary exercise testing, dedicated left and right heart echocardiography, lung ultrasonography, and cardiac MRI.

**Results:**

Our results demonstrate that subjective quality of life after COVID-19 (EQ-5D visual acuity scale, VAS, 67.4 for patients treated as outpatient, 79.2 for patients admitted to the general floor, 71.8 for patients treated in an ICU) is not related to the severity of the initial infection. Maximal exercise capacity is also reduced (VO_2_max 79% predicted, SD ± 19%); however, this is driven in large parts by patients who had initially required ICU-level of care. The degree of objective reduction in exertion did not correlate with quality of life scores. Pulmonary function testing revealed mild and persistent reduction in D_LCO_ over the first 12 months without significant restrictive or obstructive lung disease. Left and right heart function was intact with good RV function and intact RV/PA coupling, imaging findings suggestive of myocarditis were uncommon (7% of patients).

**Conclusion:**

A reduction in exercise capacity after COVID-19 is common, but is most prominent in patients previously treated in the ICU and more likely related to deconditioning or fatigue than to cardiopulmonary impairment. Subjective quality of life scores are independent of the severity of initial infection and do not correlate with objective measures of cardiopulmonary function. In our cohort, persistent cardiopulmonary impairment after COVID-19 was uncommon. The post-COVID syndrome is unlikely to be the result of cardiopulmonary sequalae and may reflect a post-ICU syndrome in some.

*Trial registration* Registered on clinicaltrials.gov (NCT04442789), Date: June 23, 2020

**Supplementary Information:**

The online version contains supplementary material available at 10.1186/s12931-022-02173-9.

## Background

The severe acute respiratory syndrome coronavirus 2 (SARS-CoV-2) has, to date, infected over 300 million people worldwide and has caused at least 5.4 million deaths since the first case was described in December, 2019 [1]. Data estimates indicate that the incidence and true death toll may, in fact, be considerably higher [2]. The burden of COVID-19 also extends beyond the initial infection, as an increasing body of literature demonstrates that symptoms and impairment may persist for a considerable time beyond convalescence from the acute illness (recently termed post-COVID-19 syndrome or “long COVID”). Common persistent symptoms after recovery include fatigue, dyspnea, and pain [3]. Symptoms that persist beyond 12 weeks from COVID-19 infection may be used as a case definition for the post-COVID syndrome [4]. Interestingly, the subsequent occurrence of disability is not restricted to patients with severe or critical acute illness as those with mild initial infections frequently also report persistent symptoms. Recent data demonstrated that “long COVID” was self-reported in 11.7% of 26,922 survey participants in the UK, indicating a significant burden of disease [5]. In light of the pandemic nature of COVID-19, the post-COVID syndrome may place a significant strain on already overstretched healthcare systems. Especially given the uncertainty about the disease pathology, it remains unclear how to best integrate care for post-COVID patients in the ambulatory and specialty clinic setting and how to identify patients with cardiopulmonary limitations that may require dedicated evaluation and follow-up.

Previously, abnormalities on cardiac imaging, lung imaging, and pulmonary function tests (PFTs) have been detected in COVID-19 survivors [6–9]. In particular, a sustained impairment in the diffusion capacity for carbon monoxide (D_LCO_) has been reported in several cohorts of recovered COVID-19 patients. However, patient reported symptoms appear to correlate poorly with objective measures of gas exchange in this patient population. Similarly, objectifiable impairment on cardiopulmonary exercise testing (CPET) has been multifactorial and deconditioning appears to play a role in exercise limitation [10–15]. Some of these findings may not be unique to COVID-19, as similar limitations have been previously seen in ARDS and post-ICU patients [16–19]. It may be difficult to disentangle symptoms that are unique sequelae from COVID-19 from the post-ICU or post-hospital syndrome as well as from the psychological stressors. Recent data suggest that such symptoms may also occur as post-influenza syndrome, raising the question whether there is indeed a unique post-COVID entity [20]. As previous studies have focused on individual components of cardiopulmonary and exercise function after COVID-19, we sought to comprehensively characterize patients after COVID-19 with multimodal assessment of lung and cardiac function, quality of life, laboratory analysis, and exercise testing in order to assess and correlate subjective and objective impairment, understand the time course of limitation and recovery, and to guide further strategies in the management for these patients.

## Methods

In the post-COVID-19 clinic at the Universities of Giessen and Marburg Lung Center (UGMLC) in Giessen, we have been offering follow-up to patients aged 18 years and older at 3, 6, and 12 months after a COVID-19 infection, with a focus on patients with persistent symptoms. Patients hospitalized in our clinic were offered follow-up visits in our outpatient clinic. Non-hospitalized patients were sent by their general practitioners for evaluation. Patients were assessed by clinical examination, echocardiography, lung ultrasonography (US), chest radiography or computed tomography of the chest as clinically indicated, PFTs, and CPET. Select patients underwent cardiac MRI imaging. Patients could consent to allow their data used for research purposes and were included in the analysis. The study was observational in nature. The project was reviewed and is covered under the University of Giessen ethics committee decision (AZ 58/15). Lung US was performed in a standardized fashion and the B-line pattern was classified semi-quantitatively according to severity (0: A-line pattern, 1: scant B-lines, 2: moderate B-lines, 3: marked/confluent B-lines, and 4: consolidation, see supplement). For secondary analysis of those cases where LVEF was not available from dedicated left heart imaging, E-point septal separation (EPSS) was measured in the parasternal long axis view and the LVEF calculated according to the following formula: LVEF = 75.5 – (2.5 × EPSS in mm) [21]. MR Imaging was performed at a 1.5 Tesla system (Somatom Avanto, Siemens Healthineers, Forchheim, Germany) using a six-element phased array cardiac coil with a standardized MR imaging protocol containing localizer/scout images, CINE imaging with steady-state free precession sequences (SSFP) obtained during breath hold (aligned to long-axis in 2-, 3- and 4-chamber view and to short-axis), native T1 and T2 mapping, late gadolinium enhancement (LGE) imaging (T1 gradient echo with inversion recovery) acquired 12 min after contrast agent injection. As contrast material Gadobenate dimeglumine (Gd-HP-DO3A; ProHance, BRACCO Imaging) was injected at a dose of 0.15 mmol/kg. Postprocessing was performed by using the cardiovascular imaging software version 42 (Circle Cardiovasculare Imaging, Calgary, Alberta, Canada). LV and RV volumes were calculated via semiautomated definition of endocardial borders on short-axis CINE stacks. Native T1 times and T2 times were measured for the septum at basal short-axis or midventricular short-axis section in regions of interest (ROIs) as proposed in the clinical recommendations by the Society for Cardiovascular Magnetic Resonance (SCMR) and European Association for Cardiovascular Imaging (EACVI) [22]. All ROIs were drawn carefully and software assisted by predefining an epicardial and endocardial offset of 10% to avoid measuring of partial volume-averaging artefacts and registration errors with gradual T1 changes at myocardial borders. Diagnostic of myocarditis more specifically COVID-19 associated myocarditis was based on the established revised Lake-Louise criteria for non-ischemic myocardial inflammation published in 2018 [23]. Lung ultrasound and CPET interpretation were performed by separate independent observers. For CPET, patients underwent a symptom-limited incremental CPET (Vmax 229 system, Vyaire Medical, Mettawa, IL, USA) using a cycle ergometer in a semi-supine position. Initial baseline values were obtained for 2 min after which a graded exercise protocol was initiated at a work rate of 10–30 W which was increased by 10-30 W every 1–2 min in a stepwise fashion [24]. For lung ultrasound, the intraclass correlation coefficient for intra-rater and inter-rater reliability were 0.987 and 0.962, respectively. Pulmonary function testing was performed in the Universities of Giessen and Marburg Lung Center (UGMLC) Giessen PFT laboratory in conformity with the European Respiratory Society/American Thoracic Society technical standards.

Statistical analysis was performed using JASP Version 0.14.01. Common statistical tests such as Analysis of Variance (ANOVA), Pearson correlation coefficient (correlation, for quantitative variables) and Spearman rank correlation (correlation, for ordinal variables) were used and are listed in the relevant sections of the text and figure legends. A p-value of < 0.05 was used to denote significance.

## Results

**Fig. 1:**
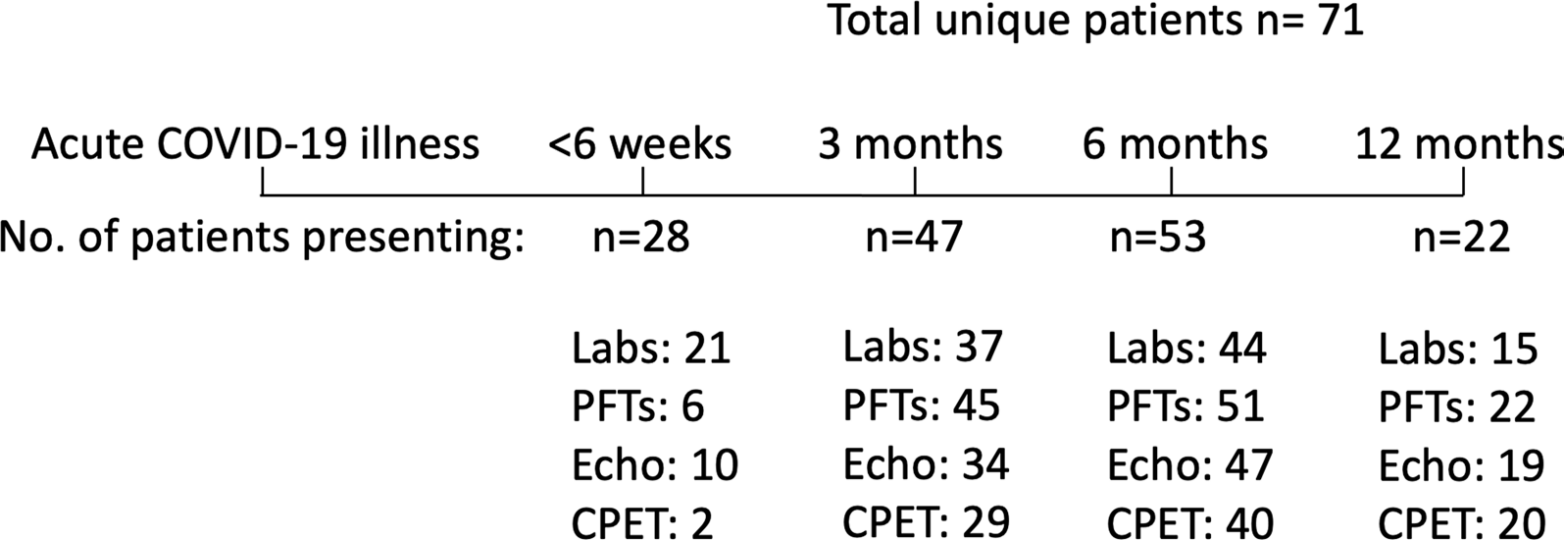
A total of 71 patients were seen for at least 1 follow-up encounter. The number of patients that showed up for each follow-up appointment is listed as well as the number of patients who underwent individual testing at the predefined intervals

In our longitudinal post-COVID clinic, 71 patients (46 males, 25 females) consented to participation (DZL, German Center for Lung Research, database and biobank for research purposes, NCT04442789). The mean age at initial infection was 56 years (SD ± 12.4 years). 21 patients had been treated as outpatients, 24 patients had been treated on a general medical floor, and 25 patients had been treated in an intensive care unit (the treatment setting of 1 patient was not ascertainable). Out of these, 8 patients underwent non-invasive ventilation and 13 patients received invasive mechanical ventilation. The mean baseline Charlson Comorbidity Index was 1.9 (SD ± 2.2). A total of 150 encounters were available for analysis, an overview of the measurements available at the pre-defined intervals is available in Additional file 1 (Fig. 1).

**Fig. 2:**
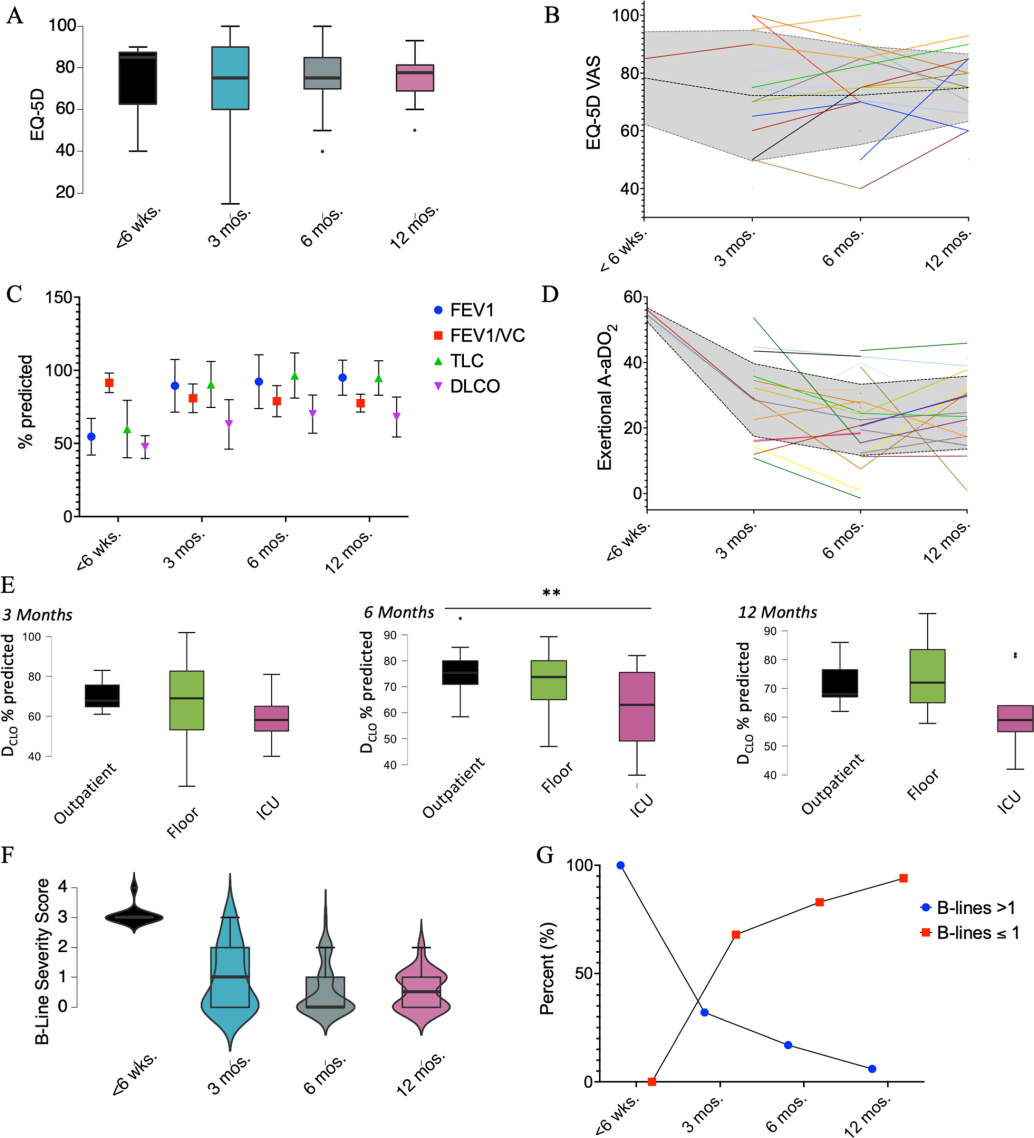
**A** EQ-5D Visual Analog Scale (VAS) over time. **B** Spaghetti plot of individual EQ-5D scores over time, dotted line represents the mean over time, shaded area represents ± 1 standard error. **C** Selected pulmonary function test results over time (blue dot – FEV1, red square FEV1/VC, green triangle TLC, purple triangle D_LCO_). **D** Spaghetti plot of individual exertional A-aDO_2_ values over time, dotted line represents the mean over time, shaded area represents ± 1 standard error. **E** Follow-up D_LCO_ measurements as % predicted based on initial treatment setting at 3, 6, and 12 months, ± SD. Significance assessed by ANOVA and post-hoc Tukey test. **F** B-line severity score over time, ± SD. **G** Relative distribution of significantly abnormal (B-line severity score > 1) as % over time. * p ≤ 0.05, ** p ≤ 0.01, *** p ≤ 0.001

Most patients who presented to the post-COVID clinic reported at least one symptom (91%). The most commonly reported symptom was decreased exercise tolerance (44%), followed by dyspnea (39%), and fatigue (26%) (Table 1). Quality of life was assessed by the EQ-5D questionnaire, the average visual analog scale (VAS) score was 72.8 (SD ± 17.7). The average EQ-5D VAS did not change significantly over the first year following the COVID-19 diagnosis (Fig. 2A). Patients treated in the ICU or hospital did not have a lower EQ-5D VAS compared with patients who only required outpatient treatment (average EQ-5D VAS from encounters in post-ICU patients 71.8 vs. 79.2 in post-general floor patients vs. 67.4 in previous outpatients).

**Table 1:** Patient characteristics

	Mean	SD	
Age	56 yrs	± 12.4 yrs	
CCI	1.9	± 2.24	
Gender	46 male, 25 female		
Level of care			
	Outpatient	(WHO 1–3)	21
	Medical floor	(WHO 4–5)	24
	ICU	(WHO 6–9)	25 (8 HFNC/NIV, 13 IMV)
Frequency of reported symptoms			
Decreased exercise tolerance			4%
Dyspnea			39%
Fatigue			26%
Chest pain			17%
Neurologic symptoms			14%
Dysgeusia			12%
Hair loss			6%
Other			14%
No symptoms			9%

We found a rapid recovery of lymphocyte counts at 3 months after the initial infection (Additional file 1: Figure S1). There was no significant association between lymphocyte counts (total or individual CD4 and CD8 numbers) and symptoms as measured by EQ-5D VAS or exertional capacity as measured by maximal oxygen uptake (VO_2_max).

Patients underwent serial pulmonary function testing, ideally at the pre-defined 3, 6, and 12-month intervals from initial infection. There was no indication of significant obstructive airway disease with the FEV1/VC ratio (Tiffeneau-Pinelli index) stably ranging above 70% throughout the first year on average (Fig. 2C, Table 2). The D_LCO_ was significantly reduced during at the 3-month time point (63% predicted, SD ± 16.9%) phase following infection with subsequent improvement, but remained slightly reduced throughout the first year. TLC was reduced acutely but remained within normal limits, on average, throughout the first year. In the patients (n = 6) that presented within 6 weeks of their initial infection, PFTs also revealed decreased FEV1 and FVC (54.6%, SD ± 12.5% and 46%, SD ± 13.6%) with a high FEV1/VC ratio (91.5%, SD ± 6.7%), suggestive of increased lung elastic recoil. A low TLC (59.90% predicted, SD ± 19.6%) demonstrates restriction. The D_LCO_ maneuver was only available from 2 patients at this early time point (47.5 mL/min/mm Hg, SD ± 7.8 mL/min/mm Hg). Subsequent follow-up D_LCO_ values were lower for patients who had been treated in the ICU compared with those treated as outpatients or the medical floor (Fig. 2E). Similar to the reduction in D_LCO_, the exertional alveolar-arterial oxygen gradient (A-aDO_2_) was measured and tracked over time, it was abnormal on 19 of 95 CPETs (abnormal widening defined as > 35 mmHg). The individual values were tracked over time (Fig. 2D). Beyond 6 weeks, average exertional A-aDO_2_ values were not significantly different over time (ANOVA, p = 0.155).

**Table 2:** Serial measurements

	< 6 weeks			3 months			6 months			12 months		
	Mean	SD	n	Mean	SD	n	Mean	SD	n	Mean	SD	n
*PFTs*												
FEV1	54.59	12.505	6	89.66	17.732	45	92.27	18.418	51	95.005	12.029	21
FVC	46.044	13.617	6	83.965	21.607	44	88.94	16.105	51	91.684	14.995	22
FEV1/VC	91.482	6.71	6	81.453	10.33	45	78.893	10.649	51	77.529	6.107	21
RV	86.774	44.355	6	93.254	28.799	44	100.31	33.121	51	93.044	14.318	22
TLC	59.863	19.64	6	89.027	20.146	44	96.447	15.467	51	94.796	11.811	22
DLCO	47.5	7.778	2	62.413	18.587	41	70.044	13.088	46	68.041	13.69	22
SaO2	97	1.583	6	96.616	1.643	43	96.864	1.302	50	96.914	1.44	21
paO2	85.38	31.559	6	78.591	10.515	44	81.212	9.728	49	80.66	9.751	21
*Echocardiography*												
LVEF	57	1.826	4	57.909	3.961	11	58.067	4.712	30	56	n.A	1
LVEF by EPSS	66.85	6.095	5	63.363	4.973	31	64.743	5.47	38	65.484	6.443	16
Longitudinal Strain	− 24.025	4.246	4	− 21.354	2.738	13	− 23.139	4.349	28	− 18	NA	1
TAPSE	21.87	5.503	10	21.938	3.282	34	22.649	3.329	47	22.221	2.531	19
TAPSE/sPAP	0.692	0.359	6	0.781	0.185	22	0.875	0.297	24	0.843	0.154	9

On lung ultrasound, initially all patients exhibited excessive B-lines (defined as a B-line score of > 1) if seen within 6 weeks of COVID-19 diagnosis. This dropped to 31% of patients at 3 months and further decreased to 6% after 12 months (Fig. 2F, G). The lung ultrasound score at 3 months demonstrated good correlation with the degree of gas exchange impairment as measured by contemporaneous exertional A-aDO_2_ during cardiopulmonary exercise testing (Spearman’s rho 0.738, p < 0.001) and D_LCO_ (Spearman’s rho − 0.547, p = 0.003). This association was not observed on subsequent testing, where anatomic changes seen on ultrasonography were largely resolved, while mild impairment in gas exchange (as determined by exertional A-aDO_2_ and D_LCO_) persisted and remained generally stable from 3 months out.

In the patients (n = 41) who underwent left heart echocardiography at least once, the initial 3D LV ejection fraction was 58% (SD ± 4%) with 35 patients (85%) having a normal LV ejection fraction (> / = 55%). 39 patients also underwent assessment by global longitudinal LV strain, which was normal (< − 18%) in 88% of patients, borderline (− 16 to − 18%) in 5 patients, and abnormal (> − 16%) in no patients. LV ejection fraction did not change significantly across follow-up time points (Table 2). Given the limited availability of 3D echo data at 12 months, we additionally assessed LVEF by E-point septal separation (EPSS) in those patients for whom only limited 2D echo images were available and calculated the LVEF. This also did not reveal any significant impairment or change in LVEF across the first year.

Dedicated right heart echocardiography was available for 66 patients. Mean values TAPSE values were 22 mm (SD ± 3 mm). This was consistent over time (Table 2). The TAPSE/sPAP ratio as a measure of RV-to-PA coupling was similarly within normal limits (mean 0.8 mm/mmHg, SD ± 0.25 mm/mmHg). 4 patients presented within 6 weeks of their infection and demonstrated a normal LVEF (57%, SD ± 1.8%), normal LV longitudinal strain (− 24%, SD ± 4.3%), normal TAPSE (21.9 mm, SD ± 5.5 mm), and normal TAPSE/sPAP ratio (0.69 mm/mmHg, SD ± 0.36 mm/mmHg).

In those patients who underwent cardiac MRI (n = 41), imaging demonstrated an average LV ejection fraction of 60.6% (SD ± 8.8%) with 34 patients (83%) having a normal LV ejection fraction on cMRI. Late gadolinium enhancement and regional wall motion abnormalities were uncommon, occurring in only 7% of patients. Ultimately, imaging review by a dedicated radiologist yielded an imaging pattern consistent with myocarditis in just 3 (7%) of patients, with 1 equivocal finding.

**Fig. 3:**
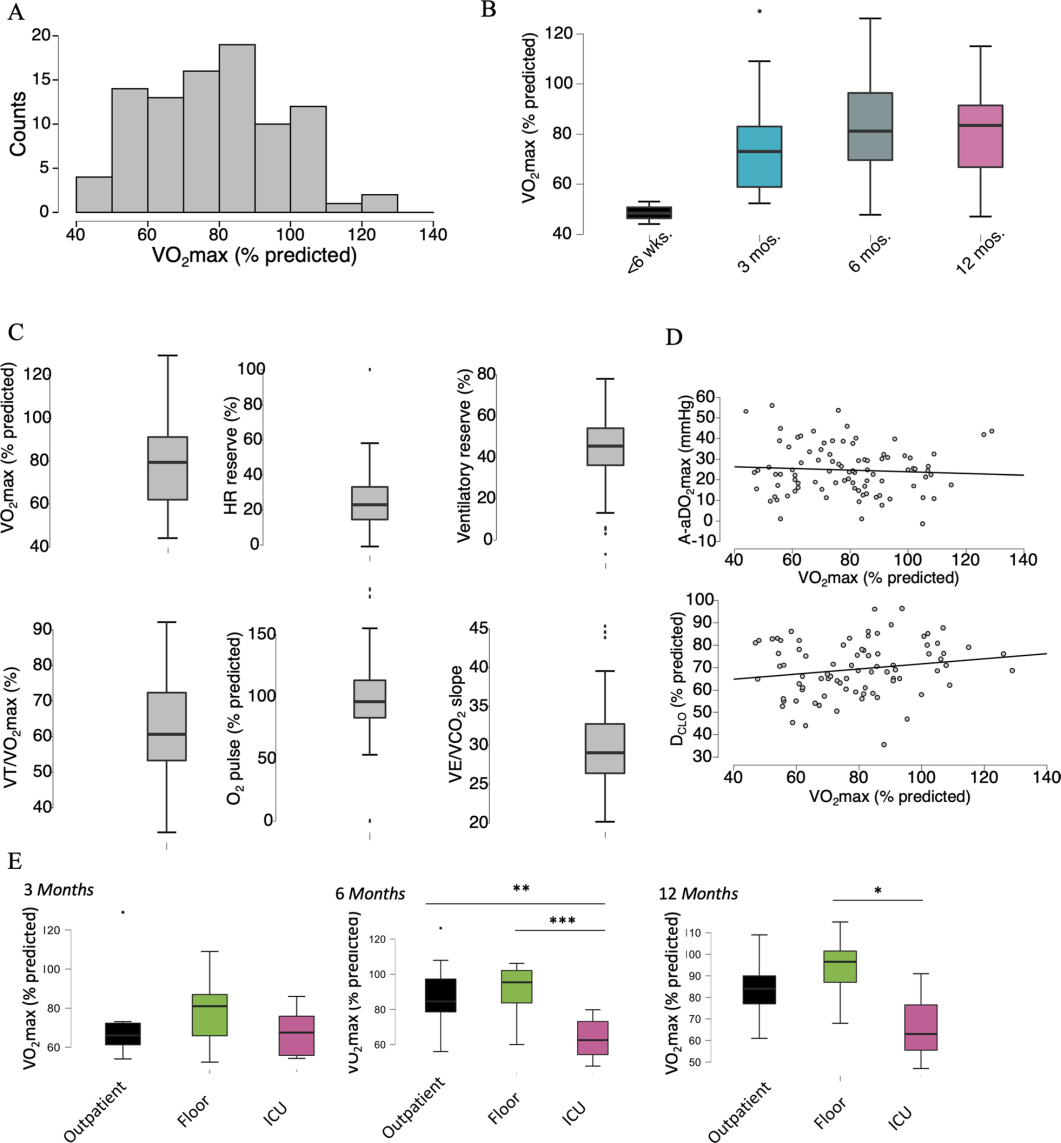
**A** Distribution of all VO_2_max values (measured in % predicted) derived from CPET. **B** VO_2_max values over time, ± SD. **C** mean select individual CPET values, ± SD. **D** Association between A-aDO_2_ on exertion and VO_2_max (top panel) as well as the association between D_LCO_ and VO_2_max. **E** VO_2_max values based on initial treatment setting over time ± SD. Significance assessed by ANOVA and post-hoc Tukey test. * p ≤ 0.05, ** p ≤ 0.01, *** p ≤ 0.001

CPET was available for 57 patients. On average, VO_2_max was reduced at 79% of predicted (SD ± 19%, Fig. 3A). VO_2_max remained mildly decreased on average across the first 12 months (Fig. 3B). The initial VO_2_max increased slightly but significantly from the 3-month (n = 29) to the 6-month (n = 40) interval (75% predicted vs. 82% predicted, p = 0.04, partially overlapping t-test). Subsequent VO_2_max values on testing beyond 6 months revealed values of 82% (9 months, n = 5) and 81% (12 months, n = 20). Similarly, while 76% of patients demonstrated an impaired VO_2_max (< 85% predicted) at 3 months, this number decreased to 60% by 6 months. Notably, at 12 months, 60% of patients still had a decreased VO_2_max. Heart rate reserve (HRR) was increased on average (25.6%, normal: ≤ 20%), while ventilatory reserve (43.3%, SD ± 16.4%), max O_2_ pulse (97.9% predicted, SD ± 26.6%), AaDO_2_max (24.2, SD ± 11.1%), ventilator threshold (63.1% of VO_2_max, SD ± 13.6%) and VE/VCO_2_ slope (29.7, SD ± 4.956) were within normal limits (Fig. 3C). Regarding only the subset of CPET studies with decreased exercise tolerance, the main limitation was again decreased HRR with preserved ventilatory reserve, O2 pulse, anaerobic threshold, and VE/VCO_2_ slope. 26.8% of patients were receiving beta blockers, however, beta blockade was more common in patients previously treated in the ICU (54%, compared with 9% of previous outpatients and 16% of previous floor patients).

In comparison, 2 patients underwent CPET testing within 6 weeks of their initial infection, at which time VO_2_max was significantly reduced (48.5% predicted, SD ± 6.4%). These two analyses demonstrated an impaired ventilatory reserve of 15% (SD ± 17%) with a HRR of 19.5% (SD ± 0.7%) and an excessively widened AaDO_2_max (54.6 mmHg, SD 2.1 mmHg), indicating a ventilatory/pulmonary pattern of limitation.

Overall, the degree of VO_2_max reduction did not correlate with the severity of gas exchange impairment as measured by D_LCO_ or exertional A-aDO_2_ (Pearson’s r 0.174, p = 0.116 and Pearson’s r − 0.064, p = 0.547, respectively, Fig. 3D). Similarly, the degree of objective reduction in VO_2_max did not correlate with the subjective quality of life of the patients (as measured by EQ-5D, Pearson’s 0.211, p = 0.084). When splitting the data based on treatment setting, it can be shown that the persistent decrease in VO_2_max is mainly driven by the patients previously treated in the ICU (Fig. 3E).

For patients who underwent contemporaneous measurement of QOL by EQ-5D and CPET (68 observations from 46 unique patients), there was no correlation between the severity of subjective symptoms and the objective exercise capacity (Pearson’s r 0.083, p = 0.499). Patients who had required ICU-level care tended to have a lower VO_2_max that persisted, but this did not correlate with a lower QOL score as measured by EQ-5D.

When restricting the scope only to those patients who had been treated in the ICU, the pattern of limitation was similar to the that seen in the overall cohort (data not shown). When plotting the VO_2_max relative to the comorbidities by CCI, there was no significant association (Spearman’s rho − 0.115, p = 0.265).

## Discussion

Post-COVID symptoms are described in a significant number of patients who have recovered from an acute infection with SARS-CoV-2. To date, the degree to which persistent cardiopulmonary abnormalities are responsible for these complaints remains poorly understood. Our study provides novel insight using a combination of pulmonary function testing, cardiopulmonary exercise testing, dedicated left and right heart echocardiography, lung ultrasonography, and cardiac MRI across multiple time points and is unique in this regard. With this battery of testing, we could demonstrate that persistent limitation in exercise capacity was common but largely restricted to patients who had previously been treated in the ICU and is not related to the degree of subjective impairment.

In line with findings from other groups, we reveal in our analyses that common persistent symptoms include decreased exercise tolerance, dyspnea, and fatigue. The fact that the severity of symptoms corresponds only poorly with the degree of cardiopulmonary impairment, seen by Townsend et al. and by our group, raises the question whether the answer is to be found in the heart/lung axis [25]. Pulmonary function testing and echocardiographic parameters did not indicate a significant or lasting impairment. The mild and persistent decrease in D_LCO_ seen after 3 months is of unclear clinical significance. It is not related to the subjective QOL scores and a persistent, mild D_LCO_ reduction has previously been described in non-COVID-19 ARDS [26]. Other groups have also seen a persistent reduction in D_LCO_ after COVID-19 [27–29].

Lung ultrasound is being used for acutely ill patients with COVID-19, where it has shown utility to assess structural lung abnormalities in a cost-effective and readily accessible manner [30]. We were able to show that lung ultrasound may be useful during the follow-up after COVID-19 as well. While abnormal lung anatomy as measured by B-lines was common at 6 weeks and 3 months, this significantly decreased over time; lung ultrasound at 3 months was strongly correlated with measures of gas exchange (exertional A-a gradient). Lung ultrasound may be used to help identify patients with persistent pulmonary impairment, objectively follow findings over time, and guide the need for further work-up after COVID-19. Our MRI data provides reassurance that protracted cardiac involvement and frank myocarditis appear to be uncommon, even after severe initial COVID-19 courses. This concern had been raised earlier during the pandemic [7], though the incidence of myocarditis varies significantly across studies [31, 32]. More recent data is in line with our findings to suggest that myocardial inflammation is present in a small number of patients after COVID-19 [33].

We also assessed quality of life scores in our patients. Although patients’ quality of life scores were not available before the COVID-19 illness for comparison, the average quality of life score of our cohort did not differ significantly from the German population norm (EQ-5D VAS average score 72.8 (SD ± 17.7) vs. 72.9 as the German population norm for EQ-5D VAS in the 55–64 age group), suggesting that the overall quality of life is not substantially different from the average person [34]. Lymphocyte counts after COVID-19 for patients in our cohort did not correlate with exercise capacity or quality of life. These findings are consistent with a previous study which also suggests that lymphocyte counts do not correlate with symptoms in patients recovered from acute COVID-19 illness [35].

Our findings additionally demonstrate that exercise limitation, as evidenced by CPET, is prevalent, especially in patients who were previously treated in an ICU. This, also, has been well described in other forms of ARDS and impairment in exercise capacity is often not explained by impairment in pulmonary function [36, 37]. General deconditioning and muscle weakness appear to be a major contributor. Akin to these findings, limitation in our patients was not related to persistent gas exchange abnormalities or pulmonary deficits. The significant heart rate reserve seen in these patients suggests that they cease exercise before reaching maximal exertional capacity. This may be related to dyspnea unrelated to underlying cardiopulmonary disease, muscle fatigue, or deconditioning. However, given the use of beta blockers in our cohort, especially in patients previously treated in the ICU, the impaired HRR may also be related to chronotropic incompetence due to pharmaceutical beta blockade.

It is important to separate the symptom complex of the post-COVID syndrome from the objectifiable cardiopulmonary impairment. Indeed, the symptoms plaguing post-COVID patients may be multifactorial in nature. One difficulty is to determine how much of the post-COVID syndrome is, in fact, novel and unique to COVID-19. Emerging evidence suggests that post-COVID-like symptoms can occur after influenza, raising the question as to the pathogen specificity of this syndrome [20]. In fact, similar symptoms have been described after various viral illnesses (including Coxsackie, influenza, and recently SARS-CoV-1) [38–40] and may reflect a broader post-viral pathology. Additionally, the psychosocial stressors of the pandemic and its far-reaching consequences including risks for job loss, financial instability, separation and loneliness may additionally contribute to the symptom complex seen after COVID-19. This shows the complexity of elucidating the pathomechanisms at play after COVID-19 and to separate the post-COVID syndrome and unique features from other post-viral syndromes.

Strengths of our study include the comprehensive nature of investigation over time, the ability to correlate various physiologic parameters among one another, and the mix of outpatient, inpatient, and critically ill follow-up cases of COVID-19. While previous studies have described individual assessments at various time points, we are not aware of integrated PFT, CPET, echocardiography, MRI, and QOL measurements over the first year. Limitations to our study include the relatively small sample size and the occurrence of missed follow-up appointments due to the number of tests. Additionally, we were restrictive with the use of chest imaging, specifically computed tomography, due to the radiation exposure. Consequently, patients were only referred for chest CT if there was a clinical indication. Our analysis does not allow to assess the influence of vaccinations as recruitment ended before the introduction of widely available vaccines. Moreover, no comparison of currently circulating SARS-CoV-2 variants is possible as recruitment ended before the emergence of the Delta or Omicron variants. However, our comprehensive analysis should make comparisons to future cohorts of patients infected with newer circulating variants possible.

## Conclusion

In summary, the symptoms and physiologic abnormalities seen after COVID-19 infection could not be correlated with a defined persistent cardiac or pulmonary organ dysfunction. Echocardiography and cardiac MRI in our cohort were reassuring, pulmonary function testing revealed a mild decrease in D_LCO_ that was most notable in patients who had required ICU-level of care, but was not correlated with exercise capacity. The decreased exercise tolerance seen on CPET, most prominently in post-ICU patients, is rather related to early termination of exercise due to deconditioning/fatigue or chronotropic incompetence. Of note, lung ultrasound may be a useful adjunct in the evaluation of post-COVID patients. Further research is urgently needed to better understand the unique contribution of SARS-CoV-2 to the post-infectious symptom complex and whether this represents a pathogen-specific process or is comparable to other post-viral or post-ICU syndromes.

## Supplementary Information

**Additional file 1: Figure S1.** A: Lymphocyte counts and subsets over time (timepoint 0: 0–6 weeks, 1: 3 months, 2: 6 months, 3: 9 months, 4: 12 months after COVID-19 diagnosis). B: Exemplary images for the classification of the predominant B-line pattern on lung ultrasound. 0: Normal pattern. Consistently thin pleural line (arrowheads) in between two rib shadows (*). A-lines apparent (arrows) in equidistant intervals (bidirectional arrows). 1: Slightly uneven and irregular illustration of the pleural line (arrowheads). Faint A-line (arrow), beginning discrete B-lines (dashed arrows), which obliterate A-lines. 2: Irregularly thickened pleural line (arrowheads). Numerous discrete B-lines (*) detectable, no A-lines depicted in this area. 3: Distinctly thickened and irregularly altered pleural line (arrowheads) depicted in between two rib shadows (+). Various B-lines (*) are seen emerging from the pleural line that radiate towards the bottom of the image, partly confluent (#). 4: Left basolateral lung zone, pleural line (arrowhead) depicted adjacent to hypoechoic, consolidated atelectasis (*) which is situated next to the diaphragm (parallel double arrow). Hyperechoic lines (arrow) within the consolidation indicate dynamic air bronchograms. Spleen (#) is visible below the diaphragm. C: Available measurements at the pre-defined timepoints.

## Data Availability

The datasets generated during and/or analyzed during the current study are not publicly available due to privacy but are available from the authors on reasonable request.

## Abbreviations

AaDO_2_max: Maximum (exertional) alveolar arterial oxygen gradient
cMRI: Cardiac magnetic resonance tomography
COVID-19: Coronavirus disease 2019
CPET: Cardiopulmonary exercise testing
D_LCO_: Diffusion capacity for carbon monoxide
EF: Ejection fraction
FEV1: Forced expiratory volume in the first second
FVC: Forced vital capacity
HRR: Heart rate reserve
ICU: Intensive care unit
LV: Left ventricle
MRI: Magnetic resonance tomography
PFT(s): Pulmonary function testing
QOL: Quality of life
SARS-CoV-2: Severe acute respiratory syndrome coronavirus 2
SD: Standard deviation
sPAP: Systolic pulmonary artery pressure
TAPSE: Tricuspid annular plane systolic excursion
TTE: Transthoracic echocardiogram
US: Ultrasound
VAS: Visual acuity scale
VE: Ventilation
VO_2_max: Maximum oxygen uptake
