# Supplementary figures and images for: Long-term comprehensive cardiopulmonary phenotyping of COVID-19
Source: Respir Res. 2022 Sep 21;23:263. doi: 10.1186/s12931-022-02173-9 (PMC9491263; doi:10.1186/s12931-022-02173-9)

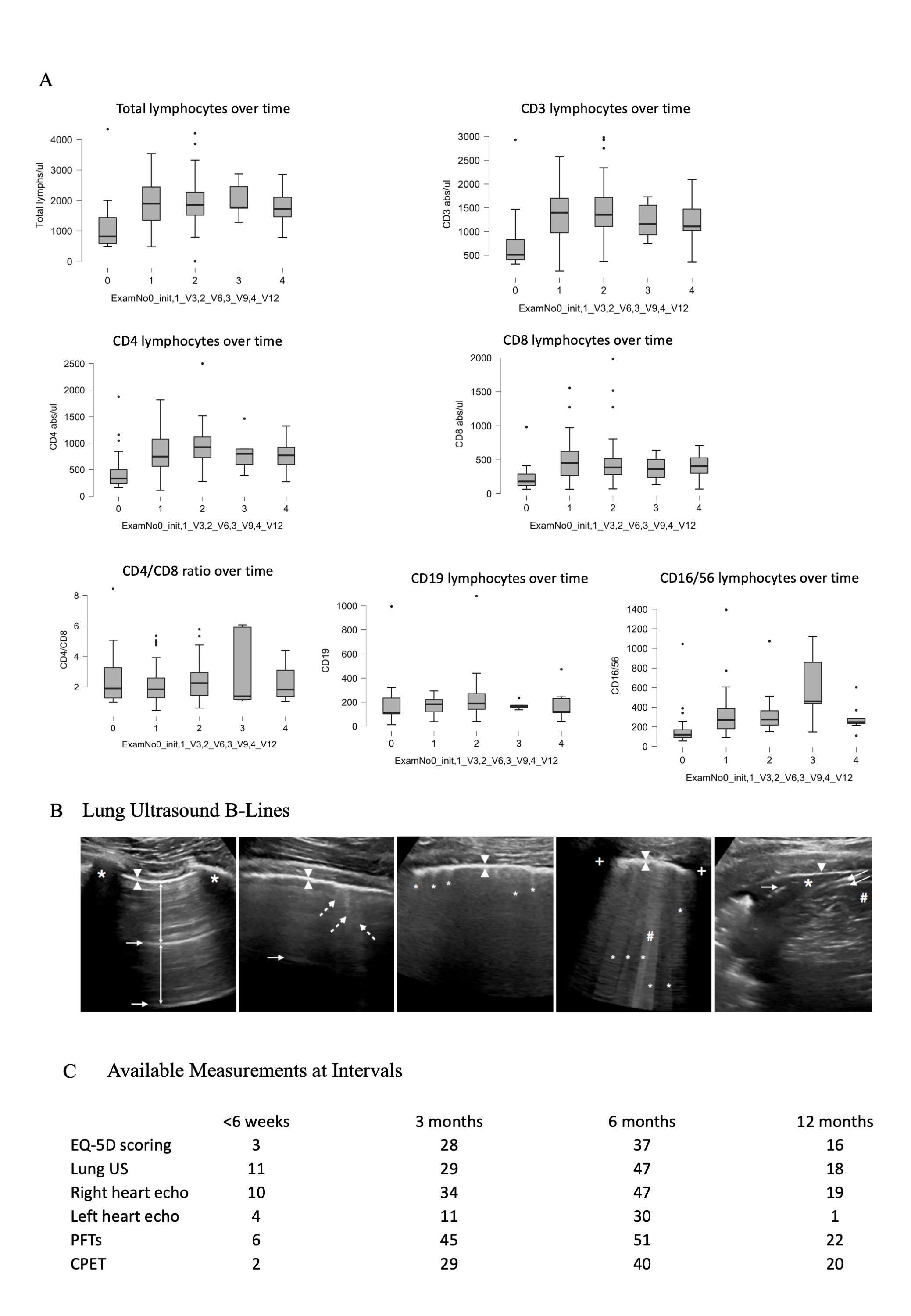

Supplement: Supplementary file 1 — Additional file 1: Figure S1. A: Lymphocyte counts and subsets over time (timepoint 0: 0–6 weeks, 1: 3 months, 2: 6 months, 3: 9 months, 4: 12 months after COVID-19 diagnosis). B: Exemplary images for the classification of the predominant B-line pattern on lung ultrasound. 0: Normal pattern. Consistently thin pleural line (arrowheads) in between two rib shadows (*). A-lines apparent (arrows) in equidistant intervals (bidirectional arrows). 1: Slightly uneven and irregular illustration of the pleural line (arrowheads). Faint A-line (arrow), beginning discrete B-lines (dashed arrows), which obliterate A-lines. 2: Irregularly thickened pleural line (arrowheads). Numerous discrete B-lines (*) detectable, no A-lines depicted in this area. 3: Distinctly thickened and irregularly altered pleural line (arrowheads) depicted in between two rib shadows (+). Various B-lines (*) are seen emerging from the pleural line that radiate towards the bottom of the image, partly confluent (#). 4: Left basolateral lung zone, pleural line (arrowhead) depicted adjacent to hypoechoic, consolidated atelectasis (*) which is situated next to the diaphragm (parallel double arrow). Hyperechoic lines (arrow) within the consolidation indicate dynamic air bronchograms. Spleen (#) is visible below the diaphragm. C: Available measurements at the pre-defined timepoints. [file 12931_2022_2173_MOESM1_ESM.tiff]
